# Characterizing Microheterogeneity in Liquid Mixtures via Local Density Fluctuations

**DOI:** 10.3390/e26040322

**Published:** 2024-04-09

**Authors:** Michael Lass, Tobias Kenter, Christian Plessl, Martin Brehm

**Affiliations:** 1 Faculty of Computer Science, Electrical Engineering and Mathematics, Department of Computer Science, Paderborn University, Warburger Straße 100, 33098 Paderborn, Germany; michael.lass@uni-paderborn.de (M.L.); kenter@uni-paderborn.de (T.K.); christian.plessl@uni-paderborn.de (C.P.); 2 Faculty of Science, Department of Chemistry, Paderborn University, Warburger Straße 100, 33098 Paderborn, Germany

**Keywords:** ionic liquids, microheterogeneity, voids, molecular dynamics, Monte Carlo, liquid phase, self organization, density fluctuations, mixtures, domain formation

## Abstract

We present a novel approach to characterize and quantify microheterogeneity and microphase separation in computer simulations of complex liquid mixtures. Our post-processing method is based on local density fluctuations of the different constituents in sampling spheres of varying size. It can be easily applied to both molecular dynamics (MD) and Monte Carlo (MC) simulations, including periodic boundary conditions. Multidimensional correlation of the density distributions yields a clear picture of the domain formation due to the subtle balance of different interactions. We apply our approach to the example of force field molecular dynamics simulations of imidazolium-based ionic liquids with different side chain lengths at different temperatures, namely 1-ethyl-3-methylimidazolium chloride, 1-hexyl-3-methylimidazolium chloride, and 1-decyl-3-methylimidazolium chloride, which are known to form distinct liquid domains. We put the results into the context of existing microheterogeneity analyses and demonstrate the advantages and sensitivity of our novel method. Furthermore, we show how to estimate the configuration entropy from our analysis, and we investigate voids in the system. The analysis has been implemented into our program package TRAVIS and is thus available as free software.

## 1. Introduction

In principle known for more than 100 years now [1], ionic liquids (ILs) are a fascinating class of organic salts with a melting point below 100 °C. They often feature advantageous properties such as low vapor pressure, low toxicity, and good thermal stability [2,3,4], and they possess a wide scope of applications in scientific and industrial fields [5,6], medicine [7,8], electrochemistry [9], as solvents [10], and in organic synthesis [3]. An important class of ionic liquids are those which are based on alkylimidazolium cations, which often feature a low viscosity, a high thermal stability, and a wide liquid temperature range [11,12,13,14]. One important feature of those compounds is the strong hydrogen bond donated by the imidazolium ring protons, which is assumed to be responsible for some of these properties and has been subject of many investigations [15,16,17,18,19]. Among the imidazolium-based ILs, special attention has been given to 1-alkyl-3-methylimidazolium salts, which have been used as solvent for biocatalysis [17], dissolving cellulose [20,21,22,23] and chitin [24], and can capture significant amounts of CO_2_ [25].

Despite being macroscopically homogeneous liquids, some ILs possess a so-called microheterogeneity or microphase separation [26]. As it is assumed that some of the advantageous properties of ILs are somehow related to such phenomena, microheterogeneity has been extensively studied both by experimental and computational approaches [2,27,28,29,30,31,32,33,34,35,36]. There exist a few computational methods for the identification of microheterogeneity in simulations of complex liquids, based on, e.g., structure factors [37], mean field approaches [38], or cluster analysis [39,40]. Another approach is the Voronoi-based domain analysis [41], which has been successfully applied to investigate complex liquids and mixtures several dozen times [29,42,43,44,45]. However, to the best of our knowledge, none of the existing approaches are well-suited to quantify microheterogeneity in ionic liquids or similar complex systems. Therefore, we have developed a new method to perform such analyses, which we present here. Our method is based on analyzing local density fluctuations within sampling spheres of varying size. It can be easily applied to both molecular dynamics (MD) and Monte Carlo (MC) simulations, including periodic boundary conditions. Our approach has been implemented into our program package TRAVIS [46,47] and is thus available as free software under the GNU GPL license.

The article is structured as follows. After a detailed discussion of the proposed method, we apply it to the simple model system of argon at different temperatures in order to introduce the different kinds of results. Subsequently, we apply our analysis to a real application—namely, simulations of the three imidazolium-based ionic liquids 1-ethyl-3-methylimidazolium chloride, 1-hexyl-3-methylimidazolium chloride, and 1-decyl-3-methylimidazolium chloride at different temperatures. We will show that our approach is well-suited to characterize and quantify microheterogeneity in these systems, and we will comparatively discuss the results obtained by the Voronoi-based domain analysis for these systems. The article ends with conclusions.

## 2. Proposed Method

**Figure 1:**
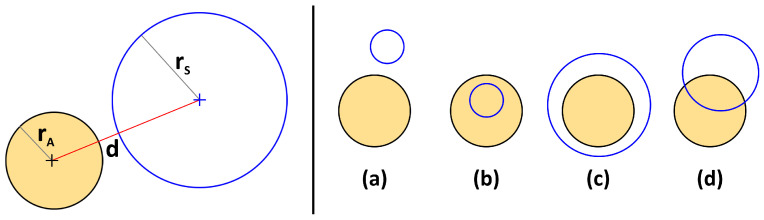
(**Left panel**) Two-dimensional illustration of the geometric situation where a spherical atom (orange-filled circle) resides next to a sampling sphere (blue circle) with radii $r_{A}$ and $r_{S}$, respectively, and distance *d*; (**Right panel**) four different cases that need to be distinguished.

Our proposed method is based on evaluating density histograms within sampling spheres of varying radius. These sampling spheres are equidistantly moved through the periodic simulation cell to obtain reasonable averaging for the histograms. Atoms in the system are not represented as points, but rather as solid spheres with predefined radii. To compute the local density within one particular sampling sphere, it needs to be computed which atoms have (possibly fractional) overlap with the sphere. The geometric situation is visualized in Figure 1. Note that the visualization is two-dimensional, while the method itself works in three-dimensional space.

In the left panel of the figure, the atom (orange-filled circle) with radius $r_{A}$ as well as the sampling sphere (blue circle) with radius $r_{S}$ are depicted. *d* corresponds to the Euclidean distance between both spheres. For each atom, we need to compute a factor *f* that describes which fraction of the atom is contained within the sampling sphere. While f=0 corresponds to the case where the atom and the sphere are disjunct, f=1 depicts the case where the atom is fully contained in the sphere. In total, four different cases need to be distinguished, which are depicted in the right panel of Figure 1. The formal criteria for these cases as well as the respective values for the *f* factors are found to be as follows (*derivation of Equation* (4): *see* Appendix A):

(a) If $d\geq r_{A}+r_{S}$:       Disjunct
  (1) $$\begin{array}{c} \;f:=0 \end{array}$$
(b) If $d\leq r_{A}-r_{S}$ and $r_{S}\leq r_{A}$:  Sphere contained in atom
  (2) $$\begin{array}{cc} & \;f:={\left(\frac{r_{S}}{r_{A}}\right)}^{3} \end{array}$$
(c) If $d\leq r_{S}-r_{A}$ and $r_{S}\geq r_{A}$:  Atom contained in sphere
  (3) $$\begin{array}{cc} & \;f:=1 \end{array}$$
(d) Else:            Partial overlap
  (4) $$\begin{array}{cc} & \;f:=\frac{1}{16\;d\;r_{A}^{3}}\cdot {\left(r_{A}+r_{S}-d\right)}^{2}\cdot \left[d^{2}+2\;d\left(r_{A}+r_{S}\right)-3{\left(r_{A}-r_{S}\right)}^{2}\right] \end{array}$$

For one particular sampling sphere with fixed position and radius, the *f* values for all atoms in the system are computed and summed up. Dividing this sum by the volume of the sampling sphere yields the particle density (unit length^−3^) inside the sphere. If the *f* value is multiplied with the mass of the corresponding atom before summing up, the mass density (unit mass^−3^) inside the sphere is obtained instead. Going one step further, the obtained density within each sphere can be divided by the overall density (either particle density or mass density) of the periodic simulation cell, leading to a relative density. Relative density values larger than 1 indicate that the density in the particular sampling sphere is larger than that of the total system (enrichment), while values below 1 depict a density in the sampling sphere smaller than that of the system (depletion).

### 2.1. Quantifying Heterogeneity

By moving the sampling sphere (*with constant radius*) to different positions within the simulation cell, one can obtain a histogram of relative densities. It immediately becomes clear how such a histogram is related to the question of possible heterogeneity in the system: In a perfectly homogeneous system, the relative density in each sampling sphere would be exactly 1, and the histogram would be infinitely narrow (*a delta distribution*) at value 1. In a real homogeneous system, there would occur some fluctuations (*e.g., due to the gaps between the spherical atoms*), but the histogram would still be relatively narrow. If the system is heterogeneous, i.e., possesses regions with different local density, the histogram will be significantly widened. The width of such a histogram can be quantified by its standard deviation σ, which can be easily computed as

(5) $$\sigma =\sqrt{\frac{1}{N}\sum_{i=1}^{N}{\left(\rho_{i}-\mu \right)}^{2}},$$

where $\rho_{i}$ is the relative density found in the *i*-th sampling sphere, *N* is the total number of sampling spheres considered, and μ is the mean value of the relative densities. Note that for a sufficiently fine-grained spatial sampling, it is always μ=1 by definition (*regions with higher-than-average and lower-than-average density cancel each other out*), so that Equation (5) can be simplified accordingly.

### 2.2. Ideal Gas as a Reference

As mentioned above, a real (*i.e., atomistic*) system which is considered to be homogeneous will still possess a relative density standard deviation σ larger than zero, because there are always gaps in a packing of spherical atoms. If we want to determine if a system is homogeneous or heterogeneous, we would require a threshold value of σ up to which we would still call the system homogeneous. Such a threshold value will obviously depend on many parameters (*sampling sphere radius, total number of atoms, …*). To find such a value, we will consider the very simple model of an ideal gas in the following. Ideal gas particles are non-interacting and do not possess a radius, and they are randomly (*uniformly*) distributed within the simulation cell. In such a situation, the average probability *p* of finding a particular ideal gas particle inside of the sampling sphere is simply given by the ratio of the sampling sphere volume and the total simulation cell volume:

(6) $$p=\frac{V_{S}}{V_{\mathrm{Total}}}=\frac{4\pi \;r_{S}^{3}}{3\;V_{\mathrm{Total}}}.$$

As the particles are indistinguishable in the scope of this analysis (*note that this is not related to the question if the particles are indeed indistinguishable in the underlying simulation model*), one can express the probability $p_{n}\left(k\right)$ to find *k* out of *n* total particles inside the sampling sphere via combinatorics:

(7) pn(k)=pk1−pn−knk=4πrS33VTotalk1−4πrS33VTotaln−knk

with the binomial coefficient

(8) nk:=n!k!(n−k)!.

Note that Equation (7) is the probability density function of the binomial probability distribution. For large particle numbers *n*, it converges towards the normal distribution, so that the histograms can be expected to look like Gaussian curves in that case (*for homogeneous systems*).

Equation (7) can be used to construct the relative density histogram for the ideal gas case, and thus also the σ value for the ideal gas. We can now introduce a relative heterogeneity measure $h_{\mathrm{rel}}$ as the quotient of observed standard deviation and ideal gas standard deviation,

(9) $$h_{\mathrm{rel}}:=\frac{\sigma_{\mathrm{Observed}}}{\sigma_{\mathrm{IdealGas}}}.$$

Values of $h_{\mathrm{rel}}$ above 1 depict some kind of heterogeneity in the system, while values below 1 indicate that the system is even more homogeneous than an ideal gas. While this may sound strange at first, it can be understood as follows. In an ideal gas, the particles do not possess a radius and are randomly distributed. In reality, atoms possess some exclusion volume and cannot penetrate each other. Due to this, less effective volume is available for the distribution of the particles, and the distribution is more regular than in the random case. Just consider a noble gas in liquid phase: The distribution of the atoms will be significantly more regular than just a random distribution of non-interacting points, leading to relative heterogeneity measures $h_{\mathrm{rel}}<1$. Please note that $h_{\mathrm{rel}}$ is a function of sampling sphere radius $r_{S}$. Larger sampling sphere radii lead to more narrow histograms—both in the real system and in the ideal gas case.

### 2.3. Estimating Configuration Entropy

One central quantity of thermodynamics is entropy. The same term is also used in information theory (*the so-called Shannon entropy* [48,49]) for a—naively seen—completely different concept. However, both concepts are closely related via statistical mechanics [50]. In the following, we will show how the local density fluctuation analysis can be used to estimate the configuration entropy of the system. Note that there are many important contributions to entropy in a chemical system, and the estimation will only cover the contribution from the distribution of the particles in the simulation cell.

For a discrete random variable *X*, the Shannon entropy H(X) is defined [50] as

(10) $$H\left(X\right):=-\sum_{x\in X}p\left(x\right)\;\log\left[p(x)\right].$$

If only a histogram of the random variable is known, the Shannon entropy can be estimated from such a histogram [51,52] by computing

(11) $$H\left(X\right)\approx -\sum_{i=1}^{n}\;f\left(x_{i}\right)\;\log\left[\frac{f(x_{i})}{w(x_{i})}\right],$$

where $x_{i}$ is the position of the *i*-th histogram bin, $f(x_{i})$ is the corresponding histogram value, and $w(x_{i})$ is the width of the bin. As we work with relative density histograms anyway, we can directly apply Equation (11) to estimate the Shannon entropy corresponding to the histogram.

### 2.4. Multiple Observations

Until now, we have considered *all* atoms in the system for computing the relative densities within the sampling spheres. However, when one speaks about a liquid possessing a microheterogeneity, one typically does not mean that the liquid possesses large voids (*filled with vacuum*). One rather refers to the fact that there is some separation of the constituents of the liquids—either the different components of a mixture, or the different regions of larger molecules, as, e.g., with tensides. In order to capture such effects, the method described above can be performed on a subset of atoms from the system, yielding *partial* relative densities and corresponding histograms, standard deviations, and heterogeneity measures. Each subset of atoms and the resulting quantities are termed one *observation*. Several such observations can be computed at the same time. An application example where this is useful will be presented in Section 4.

## 3. Verification: Argon

**Figure 2:**
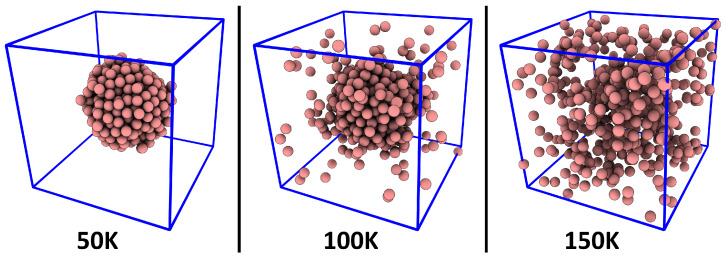
Representative snapshots of the argon simulation cell at three different temperatures.

To verify if our novel method works as intended, we have applied it to a very simple model system, namely, argon with fixed density (0.2 g cm^−3^) at different temperatures. The temperature range spans 50…300 K, so that both the liquid phase and the gas phase are captured. Due to the constant density (*which is chosen much smaller than the liquid density of* ≈1.4 g cm^−3^*, but much larger than the gas phase density of* ≈0.0016 g cm^−3^), coexistence of liquid phase and gas phase as well as aggregation phenomena can be observed at the lower temperatures. Snapshots of the simulations at three different temperatures which visually support this statement are presented in Figure 2. The computational details of the simulations are discussed in Section 5. For the analysis, the atom radius of argon (*see Equations* (1)–(4)) was set to $r_{A}=188$ pm, which is the Van-der-Waals radius [53,54,55].

**Figure 3:**
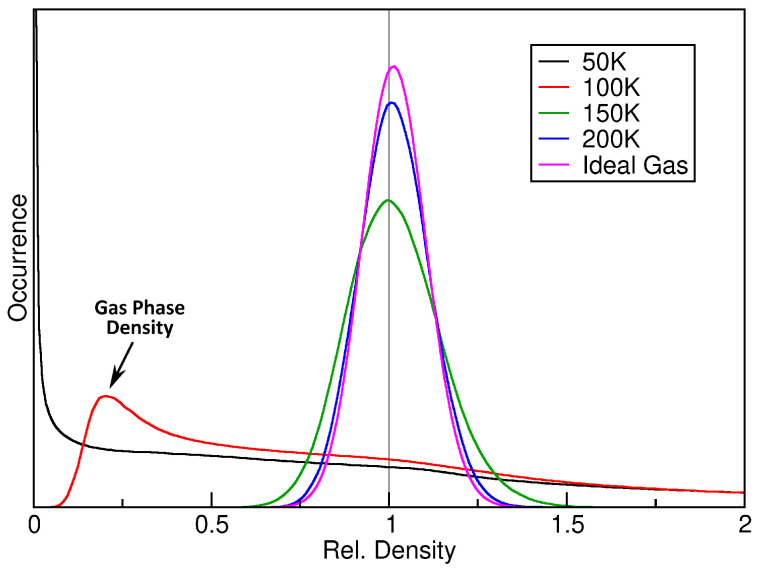
Relative density histograms of four argon simulations at a sampling sphere radius of $r_{S}=2000$ pm, together with the ideal gas histogram based on Equation (7) (*purple curve*).

As a first step, we computed the relative density histograms for a fixed sampling sphere radius of $r_{S}=2000$ pm. The results are shown in Figure 3. The black curve, which corresponds to the simulation at 50 K, shows no similarity to a Gaussian curve. It possesses a strong maximum at $h_{\mathrm{rel}}=0$, which indicates that a large fraction of the simulation cell is completely empty, because the argon atoms are exclusively found inside a liquid droplet (*see left panel of Figure 2*). When considering the simulation at 100 K, as shown by the red curve, the situation is slightly different. The single liquid argon droplet still exists, but there is now also a gas phase in the remaining parts of the cell, which stands in dynamic equilibrium with the liquid (*see middle panel of Figure 2*). Thus, the histogram has no entries at $h_{\mathrm{rel}}=0$ (*because the cell no longer possesses any empty regions*), but rather possesses a maximum at $h_{\mathrm{rel}}\approx 0.2$, which corresponds to a mass density of ρ=0.04 g cm^−3^. Therefore, the histogram clearly shows the co-existence of liquid and gas phase, and it even reveals the gas phase density in that simulation.

When considering the two higher temperatures (150 *and* 200 K, *green and blue curve in Figure 2*), the histogram becomes narrower, indicating a more homogeneous distribution of atoms in the cell, and the curve shape becomes more similar to a Gaussian function. In particular, the shape of the curve converges towards the result for the ideal gas, as derived above in Equation (7), which is shown as purple curve in the figure. This indicates that argon behaves more and more like an ideal gas at rising temperature, which is certainly true.

**Figure 4:**
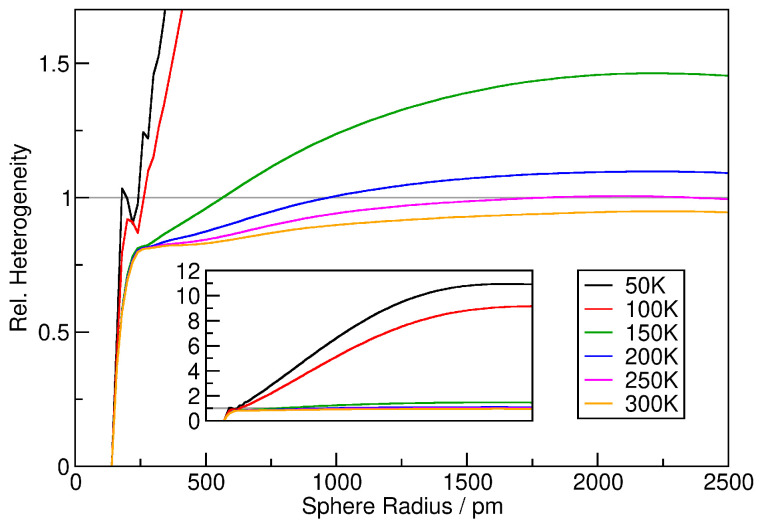
Relative heterogeneity measure $h_{\mathrm{rel}}$ as a function of sampling sphere radius for the six argon simulations. Inset shows zoom-out of the vertical axis.

To quantify the heterogeneity, it is possible to consider the relative heterogeneity measure $h_{\mathrm{rel}}$, which is related to the standard deviation of the relative density histograms relative to the ideal gas result (*see Equation* (9)). Note that this condenses the information from a whole relative density histogram into a number, so that the values of $h_{\mathrm{rel}}$ can now be visualized as a function of the sampling sphere radius (*the histograms above were only for one particular sampling sphere radius*). The results for the six argon simulations at different temperatures are presented in Figure 4. For the two simulations at 50 and 100 K (*black and red curve*), the relative heterogeneity rises fast with increasing sampling sphere radius, reaching up to values of 10 for sampling sphere radii above ≈2000 pm. This indicates that the density histogram has a standard deviation 10 times larger than that for the ideal gas, obviously caused by the phase boundary within the simulation cell (*see Figure 2*).

The four systems at higher temperatures (*150…300 K*) behave similarly up to ≈250 pm, where they all reach a plateau of $h_{\mathrm{rel}}\approx 0.8$. At this radius, the sampling sphere is only slightly larger than the argon atoms (*188 pm, see above*), so that no “soft” agglomerations can be captured. The value of 0.8 is below the ideal gas value of 1.0, which is caused by the exclusion volume of the atoms, see discussion above. If the argon atoms would be simulated as hard spheres without mutual attraction, the curves would remain at this plateau value for larger sphere radii. However, the Lennard-Jones potential that was applied accounts for attraction, and there exist fluctuating regions with slightly higher density even in the gas phase. Due to this effect, the curves begin to rise after the plateau, even crossing the $h_{\mathrm{rel}}=1$ line for 150 and 200 K. With rising temperature, this effect becomes less significant, because the attractive part of the potential is very weak and can no longer compete with the thermal motion of the atoms. We can conclude that the relative heterogeneity plots in Figure 4 give a very rich and insightful picture of the heterogeneity present in the simulations.

**Figure 5:**
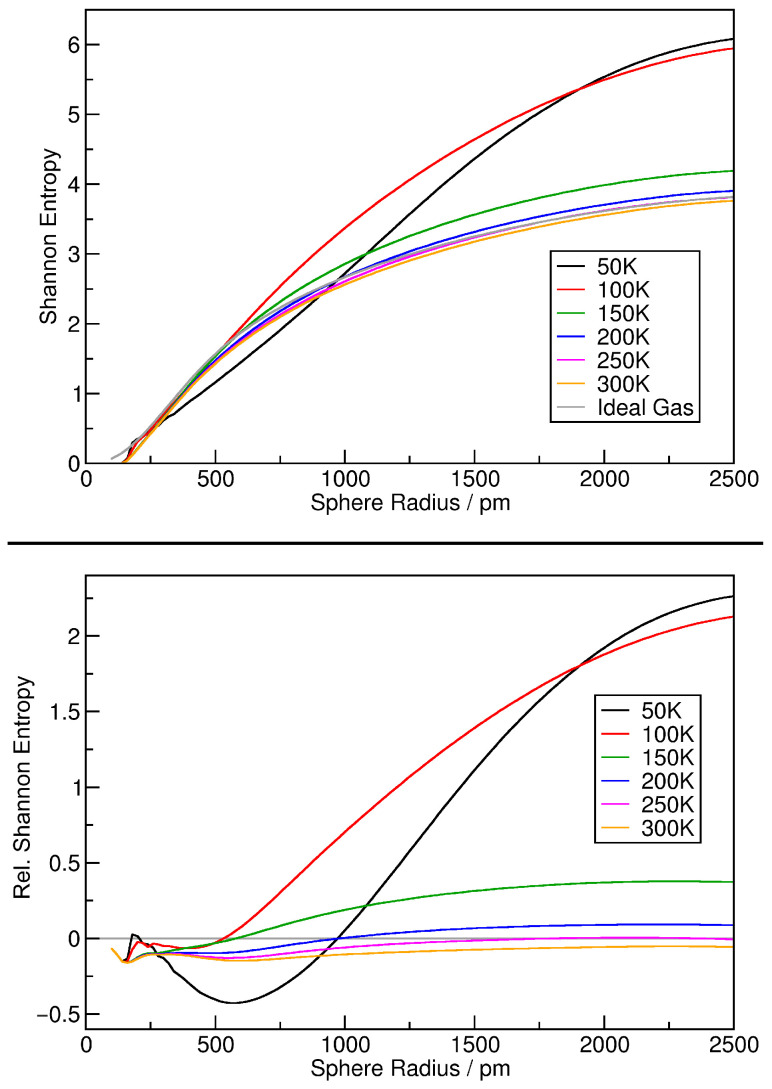
Shannon configuration entropy (*see Equation* (11)) of the argon simulations together with the ideal gas reference value as a function of sampling sphere radius (**top panel**); same results shown as relative entropies by subtracting the ideal gas reference curve (**bottom panel**).

Finally, we will estimate the Shannon configuration entropy of the argon simulations, as described above in Section 2.3. By applying Equation (11) to the relative density histograms, we directly obtain the Shannon entropy of the simulation for a given sampling sphere radius, so that we can plot the entropy as a function of the sphere radius. The same treatment can be performed for the ideal gas histogram, yielding a reference entropy function for the ideal gas case. The results are shown in the top panel of Figure 5. By subtracting the ideal gas reference function from each curve, relative entropies can be obtained, as visualized in the bottom panel of Figure 5, where the ideal gas function is indicated by the gray horizontal line. It can be seen that the entropy increases with increasing sampling sphere radius, but decreases with increasing temperature. At large temperatures, the entropy remains very close to the ideal gas curve for all sampling sphere radii. A peculiarity can be seen for the 50 K system (*black curve*) at around 600 pm sphere radius. There, the entropy drops significantly below the ideal gas reference value. This is probably a consequence of the fact that the 50 K system does not possess a gas phase—it can be considered a liquid argon droplet in vacuum.

## 4. Application: Ionic Liquids

**Figure 6:**
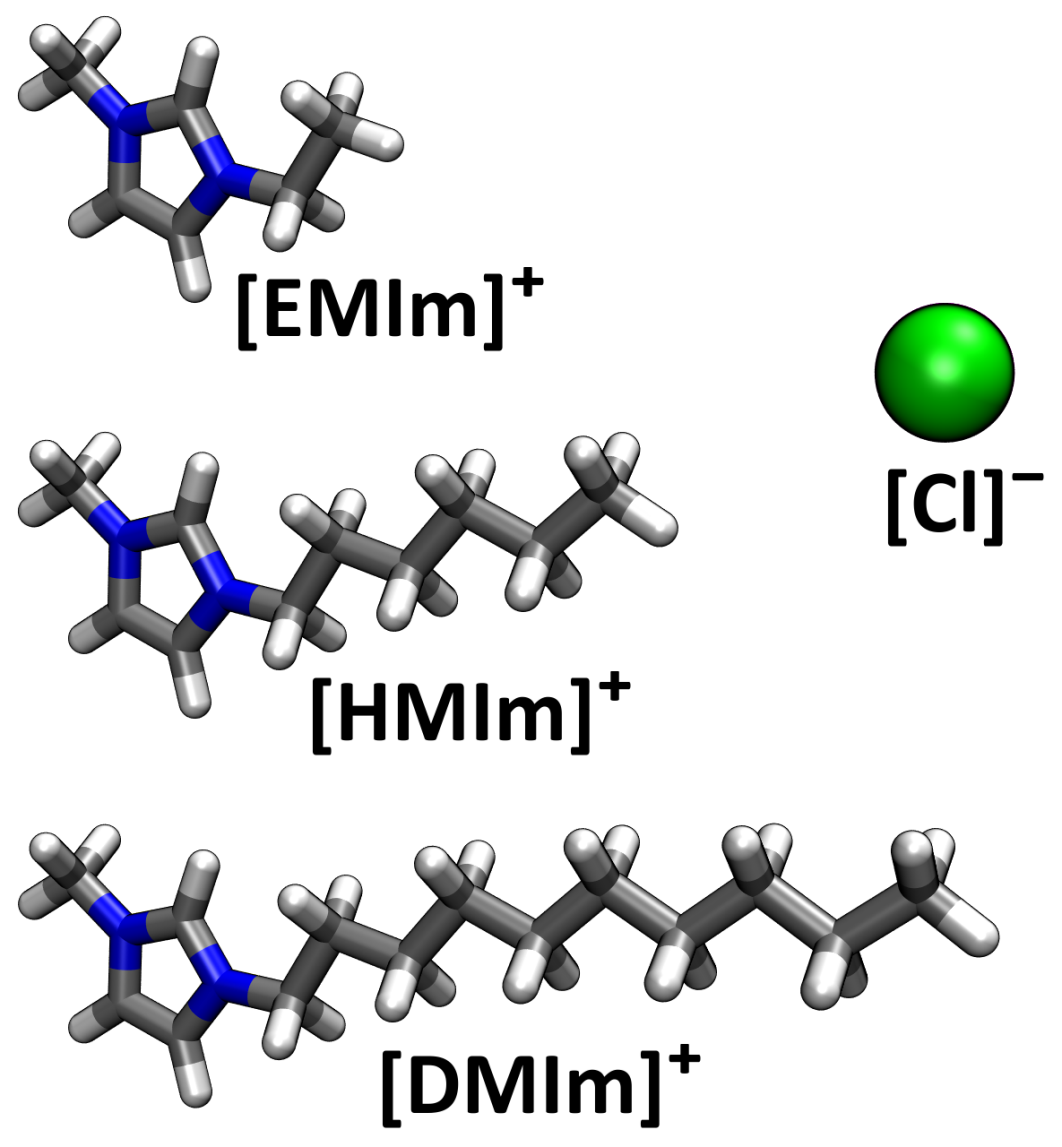
Illustration of the ions of which the ionic liquids in this study are composed.

After having validated our new method with a simple model system, we will now consider a real application—namely, three different imidazolium-based ionic liquids with different side chain lengths, each at three different temperatures. In Figure 6, the ions are shown which constitute the ionic liquids investigated here. The cations are 1-decyl-3-methylimidazolium, abbreviated [DMIm]^+^, with a side chain length of 10, 1-hexyl-3-methylimidazolium, abbreviated [HMIm]^+^, with a side chain length of 6, and 1-ethyl-3-methylimidazolium chloride, abbreviated [EMIm]^+^, with a side chain length of 2. The anion is chloride, abbreviated [Cl]^−^, in all cases. To investigate these systems, nine force field MD simulations have been performed—the Computational Details in Section 5 contain a table of the simulations.

**Figure 7:**
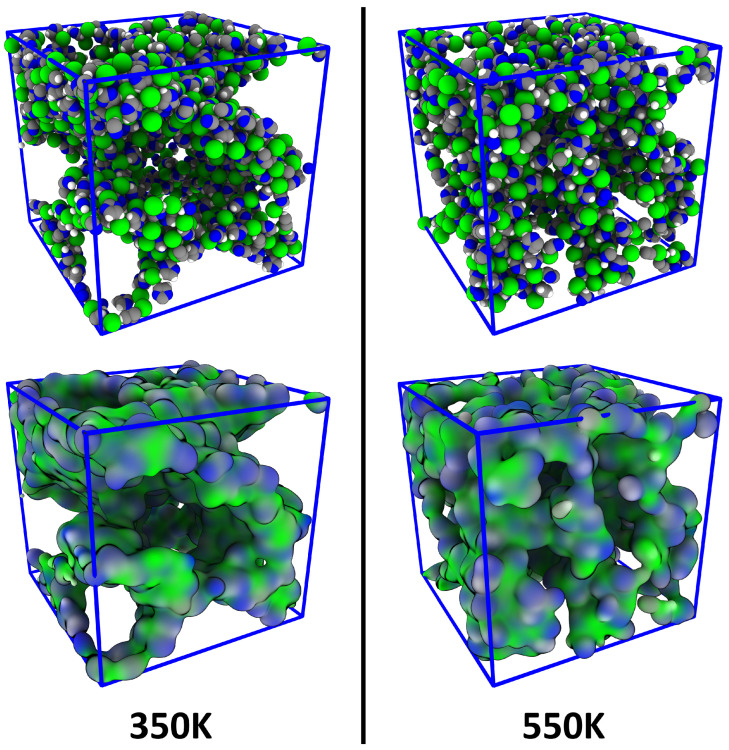
Snapshots of the simulation cell of the [DMIm][Cl] simulation—showing only the polar parts of the ions—at two different temperatures; atomistic representation (**top panel**) and surface representation (**bottom panel**). Microheterogeneity can be visually recognized.

As discussed in the introduction, it is well known that ionic liquids with relatively long side chains can exhibit a certain degree of microheterogeneity. Figure 7 illustrates snapshots from the [DMIm][Cl] simulation at 350 K (*left panel*) and 550 K (*right panel*). In the illustration, only the polar parts of the ions (*i.e., the imidazolium ring of the cations as well as the chloride anion*) are shown; the non-polar side chains are not visible. It can directly be seen that the polar parts are not homogeneously distributed in the simulation cell, and there seems to be some kind of microheterogeneity. This effect seems to be stronger at 350 K than at 550 K. However, such a statement is very vague, and a rigorous quantification would be highly desirable.

**Figure 8:**
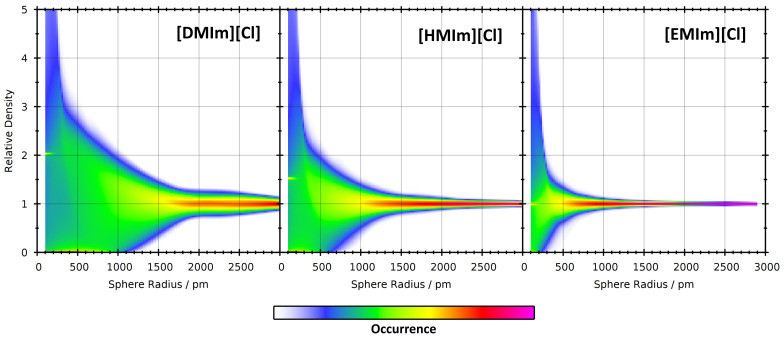
2D visualizations of the relative density histograms (vertical axis) as a function of sampling sphere radius (horizontal axis) for the three ionic liquids at 350 K.

**Figure 9:**
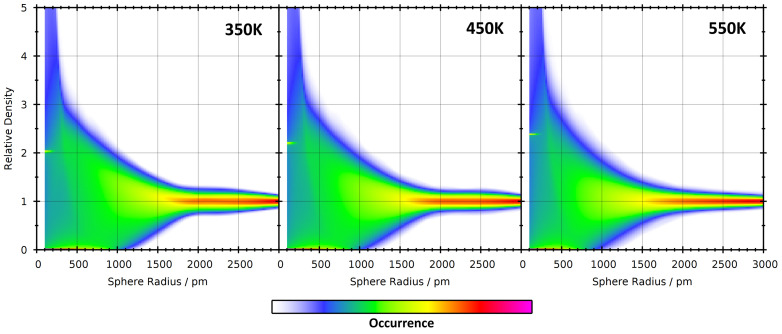
2D visualizations of the relative density histograms (vertical axis) as a function of sampling sphere radius (horizontal axis) for the [DMIm][Cl] system at three different temperatures.

The computational details of the simulations are discussed in Section 5. For the local density analysis, we have defined two observations. The first one resembles all polar parts of the system, it contains the geometric ring centers of the imidazolium cations with an “atom” radius of 300 pm as well as the chloride anions with a radius of $r_{A}=175$ pm (the Van-der-Waals radius [53,54,55]). The second observation corresponds to the terminal carbon atoms of the alkyl side chains in the cations with a radius of $r_{A}=300$ pm to implicitly account for the hydrogen atoms of the methyl group. Note that these definitions and radii concern only the trajectory analysis, but not at all the underlying molecular dynamics simulation. Many results below—e.g., Figure 8 and Figure 9—are based on the first observation (*i.e., polar parts*) only. The results for the second observation look very similar and do not add further information in these cases.

In a first step, we have computed the relative density histograms of the nine simulations. These can be visualized as 2D contour plots with the sampling sphere radius as horizontal axis and the relative density for the histogram as the vertical axis. The resulting contour plots for the three different ionic liquids at 350 K are presented in Figure 8. As expected, it can be seen that all three histograms are very wide for small sampling sphere radii, and become narrower for increasing radii. However, this trend is much faster for [EMIm][Cl] with short side chain length. This can be well understood by the following rationale. If the sampling sphere is significantly larger than the diameter of the cation, then it will automatically capture both polar and non-polar parts of the cation, and there can be no significant microheterogeneity. The largest diameter of the [EMIm]^+^ cation is around 800 pm, leading to narrow relative density histograms at radii of ≈1000 pm and above (*note that the sampling sphere size is given as a radius and not as a diameter—many [EMIm]^+^ cations fit into a sphere with a radius of 1000 pm*). [HMIm]^+^ cations are already considerably larger, with a largest diameter of around 1300 pm, and therefore the histograms remain wide up to larger radii. [DMIm]^+^ cations are very large and possess a maximum length of around 1850 pm. In that case, the histogram remains relatively wide up to very large radii of ≈2500 pm, and a significant microheterogeneity can be observed up to these length scales.

To investigate the influence of the simulation temperature on the histogram shapes, a 2D representation of the relative density histograms as a function of sampling sphere radius for [DMIm][Cl] at three different temperatures is shown in Figure 9. Already at first sight, it is visible that the three plots are very similar. There are minor differences, such as, e.g., the histogram width at $r_{S}=2500$ pm, but it would be tough to draw any quantitative conclusion from comparing these figures.

**Figure 10:**
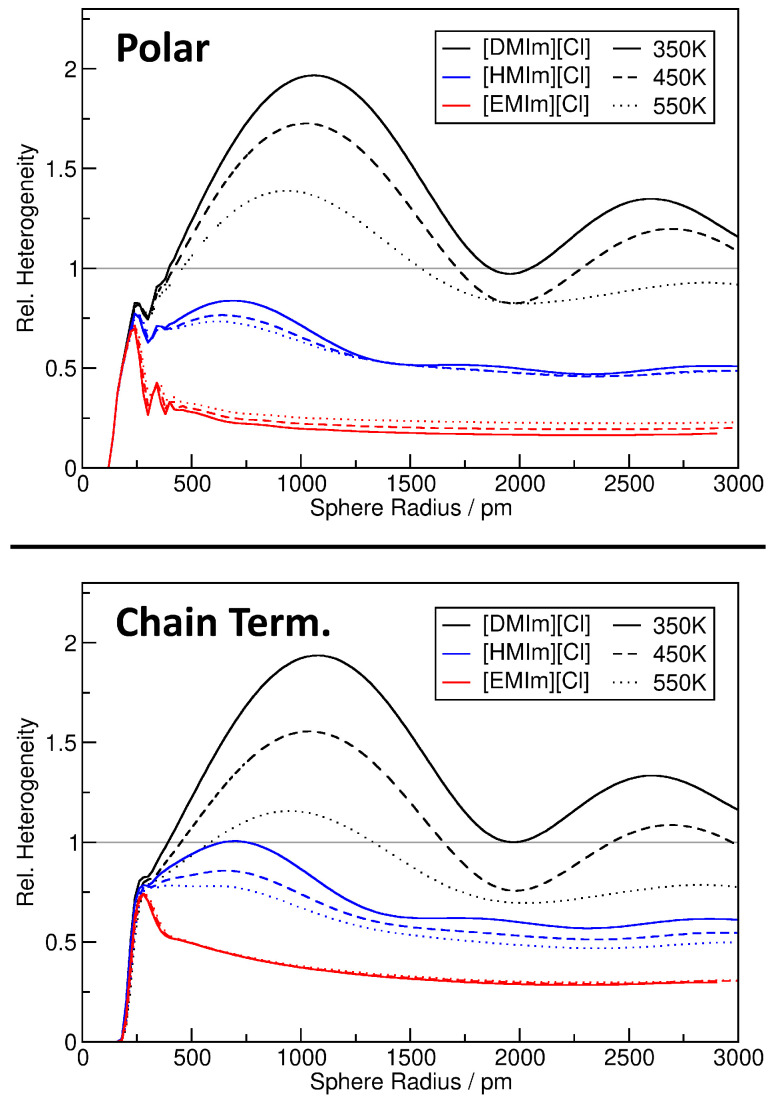
Relative heterogeneity measure $h_{\mathrm{rel}}$ as a function of sampling sphere radius for the nine ionic liquid simulations, observing either polar parts of the system (**top panel**) or chain terminal carbon atoms of the cations (**lower panel**). Grey line corresponds to ideal gas result.

For a better quantification of the heterogeneity in the nine ionic liquid simulations, we will resort to the relative heterogeneity measure $h_{\mathrm{rel}}$ as described above. The results are presented in Figure 10 for the polar parts of the system (*top panel*) as well as the chain terminal carbon atoms (*bottom panel*). Several trends are visible. First, we note that the results for both observations (*top and bottom panel*) are very similar, so that we discuss only the top panel. For [EMIm][Cl] (*red curves*), the relative heterogeneity reaches a plateau already at $r_{S}\approx 500$ with a value of $h_{\mathrm{rel}}\approx 0.25$, which indicates significantly more homogeneity than the ideal gas. This can be understood from the fact that the ions are rather small and completely fill the cell without larger voids, so that the distribution is much more ordered than just random positions (*ideal gas*). Furthermore, there is only a very weak temperature effect for [EMIm][Cl]—the systems become slightly less homogeneous at increased temperature.

The other extreme is represented by [DMIm][Cl] (*black curves*). The relative heterogeneity does not reach a plateau here, but quickly rises up to $h_{\mathrm{rel}}\approx 2$ at slightly above 1000 pm sphere radius, just to fall back to $h_{\mathrm{rel}}\approx 1$ when the sphere radius is further increased. A very interesting feature of these curves is the local minimum at around 2000 pm. The [DMIm][Cl] systems are obviously very heterogeneous, but with this particular sampling sphere radius, they appear homogeneous. The reason is the maximum length of the [DMIm]^+^ cation of around 1850 pm. Imagine a micelle-like arrangement of the ions, where the polar groups stick together, and the non-polar side chains extend to the outside in both directions. A sampling sphere with a radius of 2000 pm would exactly capture such a “micelle”, and this match in size might lead to the appearance of supposed homogeneity. Further investigation would be required to substantiate this claim, but at least it is one plausible explanation. The temperature dependence in [DMIm][Cl] is very strong, and the system becomes significantly more homogeneous with rising temperature. This is due to the fact that at higher temperature, the stronger thermal motion of the ions can overcome the energetically favored arrangement with separated polar and non-polar domains. Note that in [EMIm][Cl], the (weak) temperature dependence was of the opposite trend. Finally, we discuss the [HMIm][Cl] simulations (*blue curves*). As already expected from the molecular structure, the behavior is in between [EMIm][Cl] and [DMIm][Cl]. The systems are less homogeneous than [EMIm][Cl], but still more homogeneous than the ideal gas for all sampling sphere radii. Again, we find local minima at around 1400 pm, which fits well to the largest diameter of the [HMIm]^+^ cation of around 1300 pm and further strengthens the claim from above. The temperature dependence is significantly weaker than in the [DMIm][Cl] case, but still in the same direction (*increasing temperature increases the homogeneity*).

We can conclude that the relative heterogeneity measure $h_{\mathrm{rel}}$ is a versatile tool to understand and quantify heterogeneity in complex systems. For example, consider the temperature dependence of the heterogeneity in [DMIm][Cl], while in the discussion of Figure 9 we found that the 2D plot is very similar for the three temperatures, the $h_{\mathrm{rel}}$ plot in Figure 10 clearly shows the differences in direct comparison. Even some features such as a micelle-like arrangement of the cations can be estimated from the position of the local minima in the curves.

**Figure 11:**
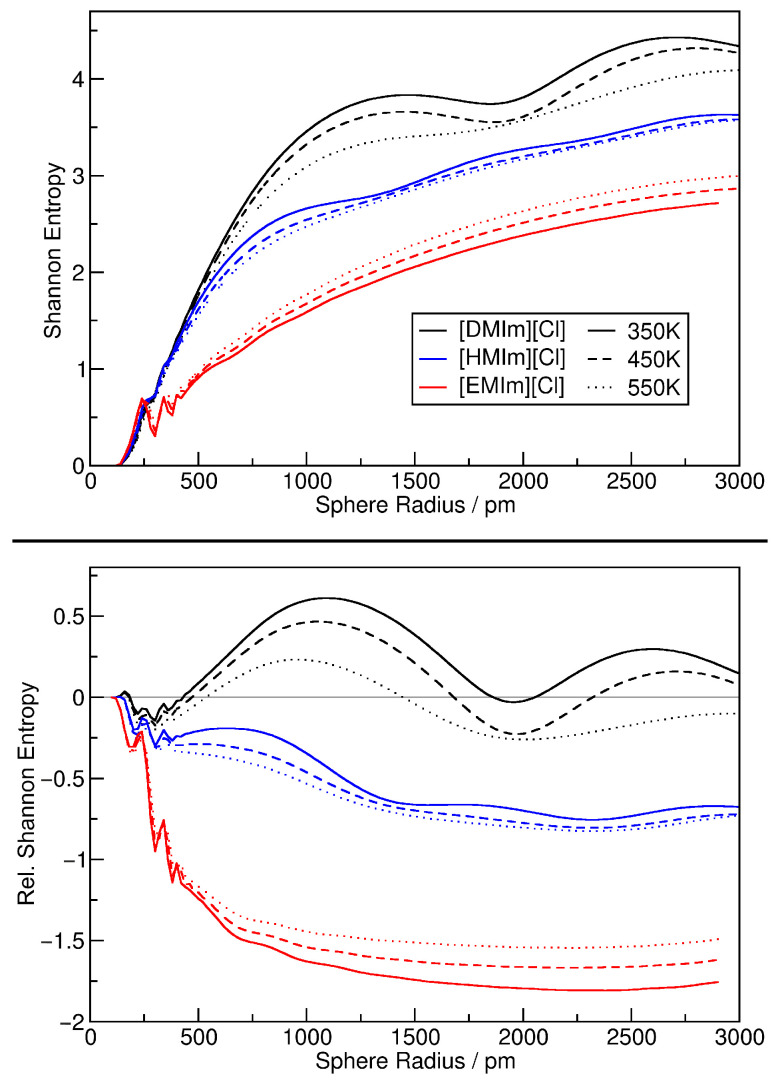
Shannon configuration entropy (*see Equation* (11)) of the ionic liquid simulations as a function of sampling sphere radius (**top panel**); same results shown as relative entropies by subtracting the ideal gas reference curve (**bottom panel**).

In a next step, we discuss the Shannon configuration entropy that can be calculated from the relative density histograms as described in Section 2.3. The results are given in Figure 11, again shown as absolute values (*top panel*) as well as relative entropies (*bottom panel*) with the ideal gas reference entropy subtracted. When comparing the top panel of Figure 10 with the bottom panel of Figure 11, we note that both plots are very similar, and the discussion of the further figure above completely applies also to the latter figure here. It is not obvious that the relative heterogeneity measure and the Shannon configuration entropy contain the same information—for the argon simulations, that was not the case (*compare Figure 4 to Figure 5*).

### 4.1. Investigating Voids

**Figure 12:**
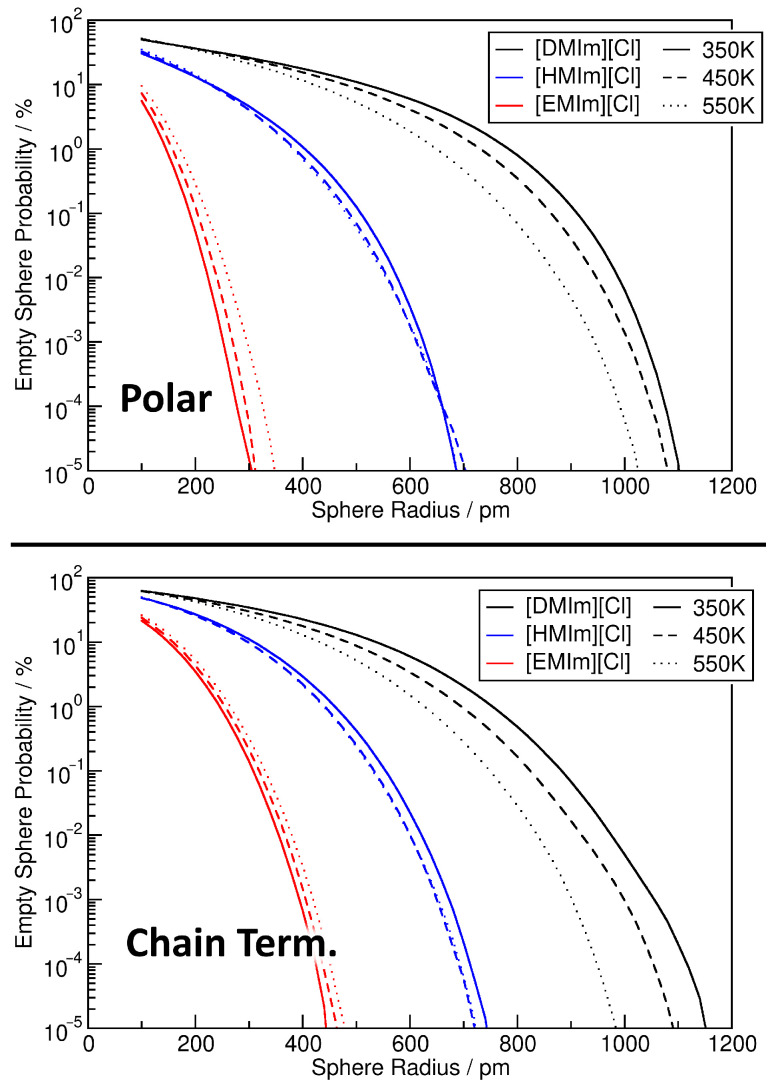
Probability of finding an empty sampling sphere as a function of sphere radius for the nine ionic liquid simulations, observing either polar parts of the system (**top panel**) or chain terminal carbon atoms of the cations (**lower panel**). Note the logarithmic vertical axis.

Another possibly interesting information which can be extracted from the relative density histograms are “voids” in the system. On the one hand side, this could be “real” voids, i.e., regions in which no atoms are located inside of the liquid. On the other hand, in microheterogeneous systems, one can also look for “partial” voids which just do not contain any atom from a subset of the system (*but other atoms instead*). For example, in a mixture, this could be a region which contains exclusively one of the constituents of the mixture. Or in systems such as [DMIm][Cl], it could be a region which does not contain any polar part of the ions. If such regions are spherical, they will be easily identified by our analysis via sampling spheres which do not contain any of the investigated atoms. The results from such an analysis are presented in Figure 12, where the sphere radius is given on the horizontal axis, and the fraction of spheres which do not contain any atom of that kind on the vertical axis (*note the logarithmic scale*). The top panel corresponds to sampling spheres which do not contain any atom from the polar part, while the bottom panel depicts spheres which do not contain any chain terminal carbon atom. As the plots corresponding to both observations are (*again*) very similar, we will only discuss the top panel corresponding to the polar part.

In the case of [DMIm][Cl] (*black curve*), relatively small sampling spheres with a radius of 100…200 pm are completely empty of polar atoms with a probability ≥50%. This percentage obviously decreases with increasing sphere radius, but is still ≈10% at $r_{S}=500$ pm, and still ≈1% at $r_{S}=750$ pm. In other words, 1% of the sampling spheres with a diameter of 1500 pm do not contain any atom from the polar part, which corresponds to a significant microheterogeneity. Going to even larger radii, we find that there even exist rare cases in which a sampling sphere of $r_{S}=1100$ pm (*i.e., 2200 pm diameter*) is completely free of atoms from the polar part. The influence of the temperature on the curves is relatively minor. As we have seen above, increasing temperature reduces the heterogeneity in the [DMIm][Cl] simulations, and therefore the probability for empty spheres is reduced with increasing temperature. When going to [HMIm][Cl] (*blue curves*), the empty spheres are significantly smaller, which is obvious from the smaller size of the cation. 1% of the spheres are empty at $r_{S}=400$ pm, and the largest appearing empty spheres have a radius of 700 pm. Finally, in [EMIm][Cl], the scale is reduced even further. Here, 1% of the spheres are empty at $r_{S}=150$ pm, and the largest appearing empty spheres have a radius of 300 pm. Despite the smaller values, this is an interesting finding. With a very small cation such as [EMIm]^+^ which has only methyl and ethyl groups as side chains, it is not obvious to find spheres of 600 pm diameter which do not contain any atom from the imidazolium ring or anion. We conclude that for systems such as ionic liquids, the sphere-based void analysis can deliver very interesting and useful insights.

### 4.2. Partial Density Correlation

**Figure 13:**
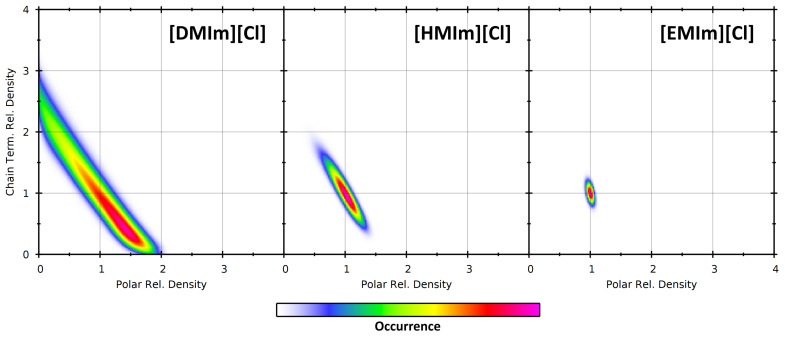
Partial density correlation histograms of the two observations (*polar parts: horizontal axis, chain terminal carbon atoms: vertical axis*) at a sampling sphere radius of 1000 pm for the three ionic liquid simulations at 350 K.

Finally, we combine the two observations (*polar parts/chain terminal carbon atoms*) and investigate the correlation between the partial densities of both observations. To do so, we compute both the relative density of polar parts and the relative density of chain terminal carbon atoms within each individual sampling sphere, and put this pair of values into a two-dimensional histogram. This reveals the (possible) correlation between both partial densities. We present the resulting 2D histograms for the three ionic liquids at 350 K in Figure 13, using a fixed sampling sphere radius of 1000 pm. The sphere radius was fixed here because a variable radius would add a third dimension and would thus complicate visualization.

For all three systems, it can be seen that there is a negative correlation: Increased partial density for one group of atoms leads to a reduced partial density of the other group of atoms. In a certain sense, this is clear, because the space in the sampling sphere is limited, and each atom from one group occupies a part of the sampling sphere which can no longer be populated by an atom from the other group. However, there can also be cases of positive correlation, such as choosing the polar parts from the cation as one observation and the chloride anions as the other. Therefore, such a correlation analysis can indeed yield additional insight into the heterogeneity in the system. As in [DMIm][Cl] the heterogeneity is strongest, the histogram peak extends up to the borders, stating that there exist sampling spheres which either include only polar parts or only chain terminal carbon atoms. When going to [HMIm][Cl] and [EMIm][Cl], this tendency decreases, because the heterogeneity and the cations themselves become smaller. But still the negative correlation persists—slightly visible even for [EMIm][Cl].

### 4.3. Comparison to Voronoi-Based Domain Analysis

There already exist other approaches which are specifically suited to investigate microheterogeneity in mixtures. One of them is the Voronoi-based domain analysis [41], which has been successfully used several dozen times in literature to investigate complex liquids [42,43,44,45]. To see if such a domain analysis can also give insight into the systems studied here, we have performed the analysis on the nine ionic liquid simulation trajectories. To obtain comparable results, the domains have been defined exactly as the observations above: one polar domain which contains the cation ring atoms (*including ring hydrogen atoms*) as well as the anions, and one non-polar domain which contains the chain terminal carbon atoms. The results are summarized in Table 1.

The first interesting quantity that results from a domain analysis is the average domain count. In the table, it can be seen that this value is very close to 1 in all cases, which means that in most of the simulation frames, all of the atoms of each group (*polar and chain terminal*) are connected via Voronoi faces—there are no isolated “islands” of either polar or chain terminal atoms. The domain count slightly rises with increasing temperature, but the difference is likely too small to be significant or meaningful. Other interesting results include the average domain surface area (*fourth column*) as well as the average domain isoperimetric quotient (*fifth column*). Both of these quantities also tend to rise with increasing temperature, but there is no way to judge which of the systems is more or less homogeneous. The strong difference in homogeneity between [DMIm][Cl] and [EMIm][Cl] can not at all be recognized from the results in the table. To summarize, we find that the results of the domain analysis are inconclusive for this particular type of system, and cannot be used to determine (*or even quantify*) microheterogeneity. This underscores the need for a generally applicable and robust analysis that works also for such systems, as the one that we have presented in this article. Note that we do not claim that our approach is generally superior to the Voronoi-based domain analysis. These two approaches are based on different principles and have their individual strengths and weaknesses, so that they nicely complement each other.

## 5. Computational Details

A list of the force field molecular dynamics simulations performed within the scope of this work is given in Table 2.

All simulations were performed with the LAMMPS package [56] (https://www.lammps.org/index.html, accessed on 10 March 2024). For the ionic liquids, the CL&P force field was utilized [57,58,59], which is an extension of the OPLS–AA force field [60,61,62] for ionic liquids. After constructing the simulation box using Packmol [63], the following equilibration protocol was employed. If some parameters (*temperature, thermostat, …*) are not specified in a step, they are identical to those in the previous step.

1. 25 ps NVT simulation at 500 K, using a Berendsen thermostat [64] with a coupling constant of 1.0 fs.
2. 250 ps NVT simulation with temperature ramp from 500 K towards target temperature, using a Nosé–Hoover thermostat [65,66,67] with a coupling constant of 100 fs.
3. 500 ps NVT simulation at target temperature.
4. 500 ps NVT simulation, using a Langevin thermostat [68,69] with a coupling constant of 100 fs to dampen possible shock waves from the temperature ramp.
5. 500 ps NVT simulation, using a Nosé–Hoover thermostat with a coupling constant of 100 fs.
6. 1 ns NpT simulation, using a Nosé–Hoover thermostat with a coupling constant of 100 fs and a Nosé–Hoover barostat with a coupling constant of 2000 fs.
7. 1 ns NpT simulation, using a Langevin thermostat with a coupling constant of 100 fs (to dampen possible shock waves) and a Nosé–Hoover barostat with a coupling constant of 2000 fs.
8. 15 ns NpT simulation, using a Nosé–Hoover thermostat with a coupling constant of 100 fs and a Nosé–Hoover barostat with a coupling constant of 2000 fs.
9. 15 ns NpT simulation, using a Nosé–Hoover thermostat with a coupling constant of 100 fs and a Nosé–Hoover barostat with a coupling constant of 2000 fs. The average density is computed during this run.
10. 1 ns simulation to monotonously shrink/grow the simulation cell to match the target density from the averaging, using a Nosé–Hoover thermostat with a coupling constant of 100 fs.
11. 1 ns NVT simulation, using a Langevin thermostat with a coupling constant of 100 fs to dampen possible shock waves.
12. 80 ns NVT simulation (*final equilibration*), using a Nosé–Hoover thermostat with a coupling constant of 100 fs.
13. 30 ns NVT simulation (*production run*).

During the production run, the positions of the particles were written to trajectory every 1000 fs. An integrator time step of 0.5 fs was used during the whole protocol, and no bonds or angles were constrained. The cutoff radius for the short-range electrostatic and Lennard-Jones interactions was set to 1000 pm. The long-range electrostatics was treated by a PPPM solver as implemented in LAMMPS [56].

For the argon simulations, a simple Lennard-Jones model with the parameters σ=340.5 pm and ϵ=0.996 kJ mol^−1^ was used [70]. The Lennard-Jones cutoff radius was set to 1500 pm in order to account for the importance of relatively weak attractive dispersion interactions in noble gases. The computational protocol was similar to the one described above, but no NpT equilibration of the cell density was performed (*fixed to 0.2 g cm^−3^*), and the equilibration and production runs were much shorter (*10 ns in total*).

All trajectory analyses were performed with the TRAVIS program package [46,47]. For the local density analysis, 200 frames were equidistantly selected from the trajectory of the production run. The center of the sampling sphere was cycled over 150×150×150 Cartesian grid points over the simulation cell, corresponding to a grid spacing of ≈40 pm, and $3.4\cdot 10^{6}$ total sampling spheres per trajectory frame for the histogram averaging.

The snapshots of the simulation cells were created in VMD [71] using the Tachyon renderer [72] with ambient occlusion. The plots were created with Gnuplot [73] and xmgrace [74].

## 6. Conclusions

In this article, we have presented a novel approach to characterize and quantify microheterogeneity and microphase separation in computer simulations of complex liquid mixtures. Our post-processing method is based on local density fluctuations of the different constituents in sampling spheres of varying size. It can be easily applied to both molecular dynamics (MD) and Monte Carlo (MC) simulations, including periodic boundary conditions.

After a detailed discussion of the method itself, we apply it to a simple model system containing the noble gas argon to verify if it works as expected. After this has been confirmed, we investigate a more complex application with our approach—namely three imidazolium-based ionic liquids with different side chain lengths that are already known to possess a certain degree of microheterogeneity. While the direct results (the relative density histograms) are of limited use for quantification of heterogeneity, we develop some derived quantities such as the relative heterogeneity measure $h_{\mathrm{rel}}$ or the sphere-based void analysis, and we show that these are powerful and robust tools to gain insight into the heterogeneity present in a system, up to detecting possible micelle-like arrangements of the ions. Based on the density histogram, we can also estimate the Shannon configuration entropy of the systems. For comparison, we apply the already published Voronoi-based domain analysis to our simulation trajectories, and find that it is not suited to detect or even quantify the kinds of heterogeneities present here, which underscores the necessity of a new heterogeneity analysis such as the one presented here.

Our novel approach has been implemented into our program package TRAVIS [46,47] and is thus available as free software under the GNU GPL license.

This manuscript is only the first step for our method. There are already plans for future work in several directions. On the one hand, we are planning to extend the approach to also investigate the temporal development of local density fluctuations, for example by constructing a density fluctuation autocorrelation function over simulation time. On the other hand, we have plans for more effective implementations, such as to rewrite the approach as a convolution on a 3D grid and use parallelized 3D fast Fouier transform (FFT) to solve it efficiently.

## Figures and Tables

**Table 1:** Results from the Voronoi-based domain analysis [41] for the three ionic liquids simulated a three different temperatures. Q is the average isoperimetric quotient of the domains.

System	Temp./K	Dom. Count	Dom. Surface Area/nm^2^	Dom. Q
[DMIm][Cl]	350 450 550	1.009 1.021 1.061	562 604 648	0.052 0.054 0.066
[HMIm][Cl]	350 450 550	1.020 1.044 1.065	724 763 806	0.049 0.057 0.064
[EMIm][Cl]	350 450 550	1.018 1.021 1.022	930 979 1032	0.046 0.047 0.046

**Table 2:** Overview of performed molecular dynamics simulations. For the ionic liquids, the cell density was equilibrated in NpT ensemble. The structure of the ions shown in Figure 6.

System	Composition	Temp./K	Density/g cm^−3^	Cell/pm	Duration/ns
[DMIm][Cl]	512 [DMIm]^+^ 512 [Cl]^−^	350 450 550	0.935 0.863 0.796	6175 6342 6515	126
[HMIm][Cl]	640 [HMIm]^+^ 640 [Cl]^−^	350 450 550	0.978 0.910 0.845	6039 6186 6341	126
[EMIm][Cl]	896 [EMIm]^+^ 896 [Cl]^−^	350 450 550	1.078 1.009 0.943	5871 6002 6139	126
Ar	512 Ar	50 100 150 200 250 300	0.200	5538	10

**Figure A1:**
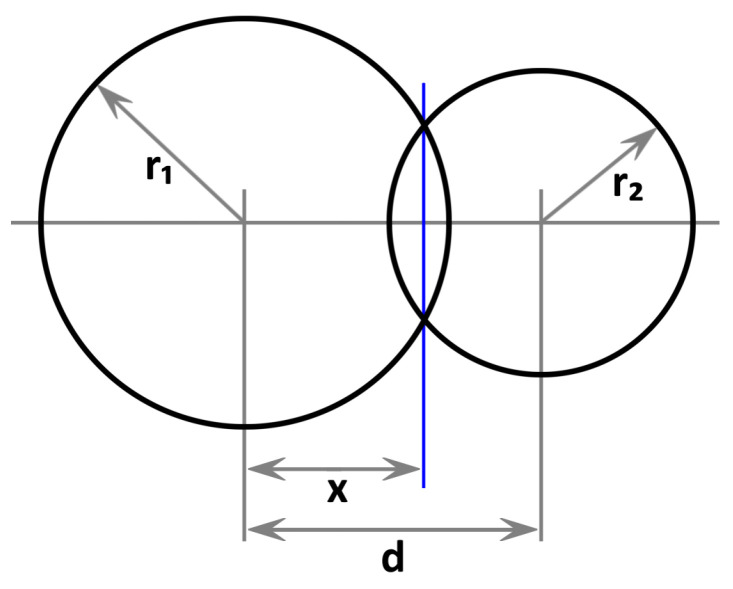
Illustration of two overlapping spheres with radii $r_{1}$ and $r_{2}$ at a distance of *d*.

## Data Availability

The software implementation of the proposed method will be made available in the TRAVIS program package, which is free software and can be obtained from http://www.travis-analyzer.de. The data from the computer simulations and the analyses can be obtained upon request from the corresponding author.

## Appendix A. Derivation of Sphere Intersection Volume

In this section, we will derive the volume of the intersection between two overlapping spheres, which is used in Equation (4). Assume two spheres with radii $r_{1}$ and $r_{2}$ in three-dimensional space. The first sphere shall be located in the origin, the second one on the X axis at position *d*. Therefore, the distance between the spheres is *d*, as illustrated in Figure A1.

The equations of the two spheres are given by

(A1) $$\begin{array}{cc} x^{2}+y^{2}+z^{2} & =r_{1}^{2} \end{array}$$

(A2) $$\begin{array}{cc} {\left(x+d\right)}^{2}+y^{2}+z^{2} & =r_{2}^{2} \end{array}$$

By combining these equations, we obtain

(A3) $${\left(x-d\right)}^{2}+\left(r_{1}^{2}-x^{2}\right)=r_{2}^{2},$$

which can be solved for *x*, yielding

(A4) $$x=\frac{d^{2}+r_{1}^{2}-r_{2}^{2}}{2d}.$$

The meaning of *x* is illustrated by the blue line in Figure A1.

To compute the volume of the intersections, we need to sum the volumes of the two spherical caps. As it can be seen in the sketch, the heights of the caps can be expressed as

(A5) $$\begin{array}{cccc} h_{1} & =r_{1}-x & & =\frac{\left(r_{2}-r_{1}+d\right)\left(r_{2}+r_{1}-d\right)}{2d} \end{array}$$

(A6) $$\begin{array}{cccc} h_{2} & =r_{2}+x-d & & =\frac{\left(r_{1}-r_{2}+d\right)\left(r_{1}+r_{2}-d\right)}{2d} \end{array}$$

The volume of a spherical cap of height *h* cut from a sphere of radius *r* is given by

(A7) $$V^{\mathrm{cap}}\left(r,h\right):=\frac{1}{3}\pi \;h^{2}\;\left(3r-h\right).$$

Now we can compute the intersection volume *V* as

(A8) $$\begin{array}{cc} V & =V^{\mathrm{cap}}\left(r_{1},h_{1}\right)+V^{\mathrm{cap}}\left(r_{2},h_{2}\right) \end{array}$$

(A9) $$\begin{array}{cc} \; & =\frac{1}{3}\pi \;h_{1}^{2}\;\left(3r_{1}-h_{1}\right)+\frac{1}{3}\pi h_{2}^{2}\left(3r_{2}-h_{2}\right) \end{array}$$

(A10) $$\begin{array}{cc} \; & =\frac{\pi }{12\;d}{\left(r_{1}+r_{2}-d\right)}^{2}\left(d^{2}+2dr_{1}+2dr_{2}-3r_{1}^{2}-3r_{2}^{2}+6r_{1}\;r_{2}\right) \end{array}$$

(A11) $$\begin{array}{cc} \; & =\frac{\pi }{12\;d}{\left(r_{1}+r_{2}-d\right)}^{2}\left(d^{2}+2d\left(r_{1}+r_{2}\right)-3{\left(r_{1}-r_{2}\right)}^{2}\right). \end{array}$$

Finally, to compute the volume fraction *f* of the first sphere which is inside of the second sphere, we divide *V* by the volume of the first sphere, obtaining

(A12) $$\begin{array}{cc} f & :=\frac{V}{\frac{4}{3}\;\pi \;r_{1}^{3}} \end{array}$$

(A13) $$\begin{array}{cc} \; & =\frac{1}{16\;d\;r_{1}^{3}}{\left(r_{1}+r_{2}-d\right)}^{2}\left(d^{2}+2d\left(r_{1}+r_{2}\right)-3{\left(r_{1}-r_{2}\right)}^{2}\right), \end{array}$$

which is equivalent to Equation (4).
